# Highway traffic flow prediction model with multi-component spatial–temporal graph convolution networks

**DOI:** 10.1038/s41598-022-18027-9

**Published:** 2022-12-14

**Authors:** Tao Ning, Yumeng Han, Jiayu Wang

**Affiliations:** 1 Institute of Computer Science and Engineering, Dalian Minzu University, Dalian, 116000 China; 2 Big Data Application Technology Key Laboratory of State Ethnic Affairs Commission, Dalian, 116000 China; 3 Institute of Computer and Communication Engineering, Dalian Maritime University, Dalian, 116028 China

**Keywords:** Nanoscience and technology, Optics and photonics

## Abstract

In order to effectively solve the problems of redundant medical material allocation, unbalanced material allocation, high distribution cost and lack of symmetry caused by unreasonable prediction in the case of sudden epidemic disasters, the prospect theory is introduced to establish a two-stage robust allocation model of medical materials, and the HQDRO based on the two-stage decision model is proposed. Aiming at minimizing the emergency response time and the total number of allocated materials, and taking the dynamic change of medical material demand in the epidemic sealed control area as the constraint condition, a two-stage robust planning model of medical materials based on scenario is established to realize the symmetrical allocation of medical materials under the sudden epidemic situation. Then, the perception model based on demand prediction, symmetry optimization, targeted distribution and psychological expectation of medical materials are constructed. Through the comparative analysis with the fitness of three commonly used algorithms in this field, the effectiveness of the robust configuration model and HQDRO proposed in this paper is verified.

## Introduction

Different kinds of serious natural disasters have occurred in many countries around the world in recent years, such as the Wenchuan earthquake in China, Hurricane Katrina in the United States, the snowstorm in southern China and, in particular, the outbreak of the New Crown Pneumonia epidemic in December 2019, which has caused great harm and losses to public health, life safety and economic and social development worldwide. During the outbreak of the epidemic, there was an explosive increase in the demand for medical supplies from countries around the world, while there was a shortage of medical supplies, ineffective connection between supply and demand information, and improper allocation of medical supplies, which reflected the existence of certain shortcomings in China's and the world's medical emergency supplies security system. The basis for good medical material security is an accurate forecast of material needs. However, the source of infection, the pathogenic mechanism of transmission, the duration, the risk of mutation, and the intensity of infection of the new pneumonia epidemic are all unpredictable, which poses great difficulties in predicting the demand for medical supplies and is also a problem that restricts the dispatch of medical supplies. In order to effectively solve the problems of difficult demand forecasting, low allocation efficiency, and high logistics and transportation costs for medical supplies, a two-stage robust allocation model for medical supplies is proposed by introducing foreground theory, while a hybrid quantum dandelion multiplication algorithm for solving the model is proposed in stage 2. Finally, the effectiveness of the proposed model and algorithm is verified through arithmetic examples. The overall structure of the paper is as follows: In Section “Current status of research”, the related works are introduced and the different methods are compared. In Section “Optimization model of medical supplies allocation”, a model of the medical supplies allocation problem under demand uncertainty is developed, describing the objective function of the problem as well as the constraints. In Section “Distribution disturbance management algorithm”, a two-stage robust configuration model for medical supplies is proposed by introducing robust control level parameters, as well as a Hybrid Quantum Dandelion Reproduction Optimization Algorithm (HQDRO). In Section “Example simulation”, the simulation examples are tested and comparatively analyzed to verify the effectiveness of the model and algorithms. In Section “Conclusion”, an improved rescheduling strategy is concluded.

## Current status of research

The key to the rational distribution of medical supplies by emergency management is to resolve the contradiction between the scarce supply of medical supplies and the excessive demand in the areas where the epidemic is being controlled. Relevant scholars at home and abroad have conducted exploratory studies on the allocation of medical supplies in epidemic areas from different perspectives, and have made progress in stages. Some scholars have developed a decision optimization model for emergency supplies allocation for public health events such as infectious diseases, using an infectious disease dynamics model to predict the demand for emergency supplies^1,2^. For example, Du et al. proposed the need for a government-led diversified medical supplies supply model by examining medical supplies security in Wuhan in response to the New Crown Pneumonia epidemic, and also proposed research frontiers in medical supplies demand to forecast and matching supply and demand for donated supplies^3^. Ge and Liu constructed a scenario for the evolution of major infectious disease epidemics in seven dimensions, including period, key events, spatial distribution and medical supplies, and proposed five key emergency supplies allocation decision problems. A multi-cycle Bayesian sequential decision model was constructed, and the validity of the model was verified by combining the Wuhan epidemic with an arithmetic analysis^4^. Fridell et al.^5^ examined the relationship between the accessibility of medical and health supplies and mortality, suggesting that effective deployment of medical supplies would reduce the impact of disaster shocks, but further evidence is needed. Barasa et al.^6^ studied the experiences of the health sector and other sectors through a literature review and summarized the factors influencing resilience in resilient organizations, including physical materials, preparation and planning, information management, subsidiary pathways and redundancy. Blanchet et al.^7^ developed a new framework for resilient governance of health systems, encompassing the ability to collect and analyze different information and knowledge, anticipate and respond to future uncertainty, manage interdependencies between environments, and develop systems and rules that conform to norms. Berardi et al.^8^ concluded through their research that diversification of the healthcare system, adequate infrastructure, and integrated emergency response have increased the resilience of the Lebanese healthcare system to cope with Syrian refugees. Qiu et al.^9^ used data from the Health Management System (HMIS) in the context of the Ebola crisis to analyze the number of indirect deaths due to lack of access to antenatal care services in Sierra Leone to assess the health system resilience. In recent years, some other scholars have predicted the number of medical supplies demanded from studies that consider the evolution of epidemics, with research methods including simulation models^10^, infectious disease dynamics models such as SIR models^11^, SIRD models^12^, IHRD models^13^, SEIR models, etc.^14^. The demand for medical supplies is mainly influenced by the evolution of the epidemic, while the supply of medical supplies also affects the evolution of the epidemic, and there is a coupling between them.

In summary, infectious disease dynamics model forecasting has been applied in the field of emergency medical supplies allocation management, but most of the relevant studies are based on macro aspects of the role of health system resilience governance strategies in the process of medical supplies allocation, while fewer studies are using medical supplies robust allocation model technology and analysis methods throughout the entire process of medical supplies allocation, and there is a lack of specific medical supplies allocation model research based on demand forecasting real-time information updates. Traditional research methods lack the technology to update material demand information in real-time, which leads to a mismatch between actual material supply and demand, inadequate targeting capabilities for allocation decisions, and insufficient medical material allocation capabilities. Therefore, there is an urgent need to combine robust techniques to study the deployment of supplies in an epidemic situation to improve the accuracy of the deployment of medical relief supplies in an epidemic.

## Optimization model of medical supplies allocation

### Problem description

Factors affecting the allocation of emergency medical relief materials include the number of emergency distribution centers and relief points, the number of emergency medical relief materials, distribution conditions and supply and demand situation, and the primary goal of allocation is to meet the needs of different relief materials in the shortest possible time. In this paper, multiple decision-making objectives such as multiple distribution centers, multiple rescue points, time cost, economic cost and equity are considered comprehensively under the condition of adequate emergency medical supplies. The problem is described as follows: after the outbreak of the epidemic, distribution points of emergency medical relief supplies of appropriate scale and quantity should be set up around the affected area, and medical supplies should be transferred from the distribution points to temporary pharmacy intravenous admixture services (PIVAS). Then appropriate distribution methods are selected to supply relief materials from PIVAS to different relief points. Assuming that each path of the road network has at least one PIVAS and *p* PIVAS exist in the epidemic area, the PIVAS set *L* can be expressed as $L = \{ L_{i} |i = 1,2, \ldots ,p\}$. The reserve amount of emergency medical relief materials of each PIVAS is *c*_1_,*c*_2_,…*c*_*p*_; There are *k* epidemic sites *R*_1_,*R*_2_,…,*R*_*k*_, the demand for emergency medical relief supplies at each epidemic point is *d*_1_,*d*_2_,…,*d*_*k*_, and $\sum\nolimits_{m = 1}^{p} {c_{m} } \ge \sum\nolimits_{n = 1}^{k} {d_{n} }$. There is a corresponding relationship between potential epidemic points and PIVAS of medical supplies, and there are *q*_*i*_ potential epidemic points for each *L*_*i*_. In the case of sufficient supply of emergency medical rescue materials, the decision objective of the allocation optimization model is to meet the demand of emergency medical aid materials of each rescue point within the specified distribution time, and to reasonably plan the distribution route, so as to minimize the total supply time of emergency medical aid materials. Make the following assumptions:

1. PIVAS can deliver supplies to relief points multiple times, and different relief supplies can be mixed.
2. The vehicles for delivering emergency medical relief materials start from the starting point and return to the starting point after completing their tasks.
3. The “demand segmentation” strategy is adopted for the massive demand rescue points, and the combination of “full load direct delivery” and “itinerant distribution” is adopted, according to the principle of full load direct delivery priority.
4. The speed of the distribution vehicle is randomly and dynamically variable.

### Symbol definition

$C_{i}$: Decision variable, indicating the material owned by PIVAS $L_{i}$.

$S_{i}^{j}$: Supplies needed for outbreak site $R_{i}^{j}$.

$s_{i}^{j}$: Distribution of medical supplies by PIVAS $L_{i}$ to point $R_{i}^{j}$ in its area of service.

$s_{i - h}^{j}$: Distribution of medical supplies by PIVAS $L_{i}$ to nearby outbreak sites $R_{h}^{j}$ outside its service area.

$r_{h}^{j}$: The urgency of the material needs of potential outbreak site $R_{h}^{j}$, $r \in [0,1][0,1]$.

$g_{h}^{j}$: Whether $L_{h}$ distributes medical supplies to $R_{i}^{j}$ during the second phase, i.e.

$$g_{h}^{j} = \left\{ \begin{gathered} 1, delivery \hfill \\ 0, no \, delivery \hfill \\ \end{gathered} \right.$$

$u_{h}^{j}$: The number of outbreaks at epidemic points $R_{h}^{j}$ during the statistical period.

$\overline{S}_{h}^{j}$: Average demand for medical supplies at epidemic points $R_{h}^{j}$ during the statistical period.

$\alpha$,$\beta$ adjustment ratio coefficient, the initial value is 0.5.

$o_{j} (0)$: In the first phase, the quantity of supplies delivered to point *j.*

$s_{j} (t)$: In the second phase, the quantity of supplementary supplies delivered to epidemic point *j* at time *t.*

$s_{ij} (t)$: Material delivered by PIVAS *i* to epidemic point *j* at time *t*.

$s_{i}^{^{\prime}} (t)$: Materiel PIVAS *i* can provide at moment *t*.

$T_{ij}$: Travel time of material PIVAS *i* to epidemic point *j*.

### Model building

A two-stage decision model can be established for medical allocation. In the first stage, the decision time and the optimal allocation of medical supplies are determined according to the characteristics of epidemic infection rate, so as to reduce the distribution cost as much as possible. In order to minimize the total allocation of medical supplies within the road network, an optimization model of medical supplies allocation is established. The second stage is to determine PIVAS and material quantity based on the optimal allocation of medical supplies, aiming at the shortest emergency delivery time. The two-stage model can be expressed as:

#### First stage model

Objective function

1 $$\min Z = \sum\limits_{i = 1}^{m} {C_{i} }$$

2 $${\text{s}}.{\text{t}}. \;\;\;\;\;\;s_{i}^{j} + \sum\limits_{h = 1,h \ne 1}^{m} {g_{h}^{j} s_{h - i}^{j} \ge S_{i}^{j} } (i = 1,2, \ldots ,m;j = 1,2, \ldots ,n_{i} )$$

3 $$\sum\limits_{j = 1}^{{n_{i} }} {s_{i}^{j} + r_{h}^{j} s_{i - h}^{j} } \le C_{i}$$

4 $$g_{h}^{j} s_{h - i}^{j} + \sum\limits_{j = 1}^{{n_{h} }} {s_{h}^{j} } \le C_{h}$$

5 $$\sum\limits_{h = 1,h \ne i}^{m} {g_{h}^{j} = 1(i = 1,2, \ldots ,m;j = 1,2, \ldots ,n_{i} )}$$

6 $$g_{h}^{j} = 0,1(i = 1,2, \ldots ,m;j = 1,2, \ldots ,n_{h} )$$

Equation (1) is the objective function, indicating that the total amount of medical supplies in the road network in the epidemic area is the least. Equations (2)–(4) represent constraint conditions, where Eq. (2) represents the quantity of medical supplies received at each epidemic point to meet the overall demand; Eq. (3) ensures that each medical supplies PIVAS has supplies that meet the needs of other potential epidemic sites; Eq. (4) ensures that each PIVAS of medical supplies can meet the demand of supplies in case of other outbreaks within the distribution range after providing the demand of supplies near epidemic points outside the distribution range in proportion. Equations (5) and (6) represent the parameter constraints of Eq. (1) and the premise of the model that only two medical supplies distributed by PIVAS can meet the rescue needs of the accident.

When an epidemic occurs, the urgency of the demand for medical supplies at potential epidemic sites depends on the frequency of the epidemic and the evaluation demand for medical supplies. The study quantified it as follows:

It is assumed that there are *n*_*h*_ potential epidemic points in *L*_*h*_ of PIVAS, $F_{j} (j = 1,2, \ldots ,n_{h} )$ represents epidemic frequency coefficient, $D_{j} (j = 1,2, \ldots ,n_{h} )$ represents epidemic material demand coefficient, then

7 $$r_{h}^{j} = \alpha F_{j} + \beta D_{j}$$

8 $$F_{j} = \frac{{\alpha_{h}^{j} }}{{\sum\limits_{j = 1}^{{n_{h} }} {u_{h}^{j} } }}$$

9 $$D_{j} = \frac{{\overline{S}_{h}^{j} }}{{\sum\limits_{j = 1}^{{n_{h} }} {\overline{S}_{h}^{j} } }}$$

#### Second stage model

In the second stage, the shortest emergency delivery time is taken as the goal. Assuming that *n* epidemic cases occur, the epidemic place is denoted by *j*, then the epidemic point collection is denoted by $R = \{ R_{j} |j = 1,2, \ldots ,n\}$. After the outbreak of the epidemic, rapid response time is the key to ensure the dispatch of emergency medical supplies, so the second phase aims to minimize the outbreak response time.

10 $$\min Z = \{ [\sum\limits_{j = 1}^{n} {(o_{j} (0) + \sum\limits_{t = 1}^{N} {s_{j} (t)} ) - \sum\limits_{t = 0}^{N} {\sum\limits_{j = 1}^{n} {\sum\limits_{i = 1}^{m} {s_{ij} (t)] + [\sum\limits_{j = 1}^{n} {(s_{j} (t) - \sum\limits_{i = 1}^{m} {s_{ij} (t)} )} ] + } } } } \sum\limits_{t = 0}^{N} {\sum\limits_{j = 1}^{n} {\sum\limits_{i = 1}^{m} {(t + T_{ij} )\} } } }$$

11 $${\text{s}}.{\text{t}}. \;\;\;\;\sum\limits_{j = 1}^{n} {s_{ij} (t) \le s_{i}^{^{\prime}} (t - 1) - \sum\limits_{i = 1}^{n} {s_{ij} (t - 1) } } t(i = 1,2,...,m;t = 0,1,2,...,N)$$

Equation (10) represents the objective function of minimizing emergency response time, and Eq. (11) represents the number of supplies PIVAS *i* provided to each epidemic point *j* at time *t* should not be higher than the total number of supplies.

### Analysis of psychological expectation perception of epidemic patients

Patients’ psychological expectation of medical supplies is related to PIVAS, patients' psychological status and the time *T*_*f*_ of the actual arrival of medical supplies. When patients eagerly look forward to a batch of medical supplies, their expectation decision weight function can be expressed as:

12 $$\tau^{i} (m(T_{f} )) > \tau^{i} (m) > 0, \tau^{i} (n) > \tau^{i} (n(T_{f} )) > 0$$

*m*, *n* represent the objective probability value of negative effects and the objective probability value of positive effects, and $\tau^{i} (m)$ represents the probability decision weight function of probability *m*; $\tau^{i} (n)$ represents the probability decision weight function of probability *n*, that is, $\tau^{i} (0)$ = 0, $\tau^{i} (1)$ = 1.

Patient’s expectation judgment of medical supplies distribution is shown in Eq. (13) :

13 $$V^{i} (T_{f} ) = \tau^{i} (m(T_{f} )) \cdot \upsilon^{i} (x) + \tau^{i} (n(T_{f} )) \cdot \upsilon^{i} (y) < \tau^{i} (m) \cdot \upsilon^{i} (x) + \tau^{i} (n) \cdot \upsilon^{i} (y) = V^{i}$$

**Figure 1:**
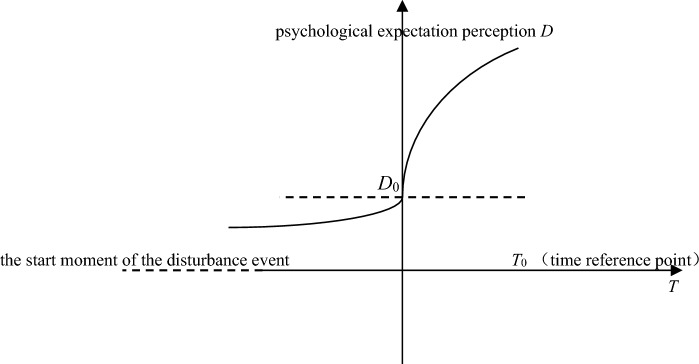
The psychological expectation perception curve chart^16^.

In Eq. (13), $\upsilon^{i}$ represents the value function, $\upsilon^{i} (x)$ and $\upsilon^{i} (y)$ represent the patient's subjective value relative to the reference point (patient's psychological expected time). If the patient's psychological expected time *T*_0_ is taken as the reference point and the prospect theory is combined^15^, when *T*_*f*_ < *T*_0_, that is, the actual arrival time of medical supplies is less than the patient's psychological expected time, the patient's psychological expected aversion degree is very small (denoting 0). When the value of *T*_*f*_ approaches *T*_0_, the patient's psychological expectation aversion increases continuously; when *T*_*f*_ > *T*_0_, the patient's psychological expectation aversion increases significantly^16^. Therefore, when PIVAS delivery time is taken as the abscissa and patients' psychological expectation perception degree is taken as the ordinate, the psychological expectation perception curve of patients for PIVAS arrival time can be deduced according to the value curve of prospect theory^17^, as shown in Fig. 1.

When *T*_*f*_ = *T*_0_, expected aversion is expressed as *D*_0_. According to the above value function model of prospect theory, the psychological expectation perception function model of patients can be set as $D^{i} (T) = - V^{i} ( - x + T_{0} ) + D^{i}_{0}$, namely, the psychological expectation perception function model of medical supplies delivery arrival time considering different disturbance target *i* is:

$$D^{i} = \left\{ \begin{gathered} - (T_{0} - T_{f} )^{{\alpha^{i} }} + D^{i}_{0} ,\quad T_{f} \le T_{0} , \hfill \\ \lambda^{i} (T_{f} - T_{0} )^{{\beta^{i} }} + D^{i}_{0} ,\quad T_{f} > T_{0} . \hfill \\ \end{gathered} \right.$$

## Distribution disturbance management algorithm

### Hybrid quantum dandelion reproduction optimization algorithm

HQDRO is a heuristic algorithm based on the floating propagation of dandelions, it does not easily get stuck at a local optimum. It has the strong robustness, however, the floating of the algorithm takes a fixed step size, it may take time to hover around the optimal location for searching and cause loss of highly adapted dandelion individuals^18^. Considering that quantum computing has exponential storage capacity, parallelism, and exponential acceleration. And the entanglement, overlap, and interference of quantum states may help to reduce the complexity of some large-scale problems^19^. So, this article proposed the HQDRO. The state and location of individual dandelions are indeterminate in quantum space, and these individuals are determined by the wave function $\psi (Y,t)$.The probability density function for individual locations is expressed as $\left| \psi \right|^{2}$, establishing the attraction on the dimension of each attractor based on the δ potential well model. The potential energy function can be expressed as follows:

$$V(Y) = - \gamma \delta (Y)$$

$Y = x_{id} - P_{d}$ is the distance between the individual position of the dandelion and its attractor.

The steps of HQDRO are described below:

Step 1: Initialize the number of dandelion individuals as *b*_*n*_, the filter times as *N*_*ed*_, the number of reproductions as *N*_*re*_, the chemotactic times as *N*_*c*_, the falling times as *N*_*s*_ and the probability of filtering as *P*_*ed.*_
Step 2: The vector *x*_*i*_ of dandelion individual *i* is randomly generated in the solution space.
Step 3: Solve the fitness function of all dandelion individuals.
Step 4: Quantum location is updated. The parameter is the transfer period *l*=1:*N*_*ed*_; the reproductive cycle *b*_*c*_=1:*N*_*re*_; the falling cycle *d*_*c*_=1: *N*_*c*_*.*
Step 5: Falling operation, adjust orientation with random vector $\Delta \in R^{n}$, each vector of $\Delta$ is a random number in the interval [− 1,1]. Update the dandelion individual position *x*_*id*_ by formula (14) ^20^, the rest of the variables remain unchanged.
  14 $$x_{id} = P_{d} \pm \frac{L}{2}\ln (1/u),u \sim U(0,1)$$
  15 $$x_{id} (d_{c} + 1,b_{c}^{{}} ,l) = x_{id} (d_{c} ,b_{c} ,l) + S(i,d_{c} )\eta (i)$$
  16 $$\eta (i) = \frac{\Delta (i)}{{\sqrt {\Delta^{T} (i)\Delta (i)} }}$$

*S*(*i ,d*_*c*_) Indicates the forward falling step size, $\eta (i)$ indicates the direction after the change.

Step 6: Filter operation. Calculate the fitness of $x_{i} (d_{c} + 1,b_{c} ,l)$. If it is better than $x_{i} (d_{c} ,b_{c} ,l)$, replace it and filter it according to the wind direction until the fitness value is stable.
Step 7: Quantum irrigation operation. Sort them according to the fitness of all dandelion individuals and irrigate the individuals (*b*, *P*_*ed*_) with higher suitability.
Step 8: Determine if the operation is done.

### Convergence comparison of algorithms

To verify the performance of the HQDRO proposed in this paper. A classical Solomon example^21^ is selected to execute one standard question for 20times from each of the six types of questions in Matlab9.0 by using HQDRO. The results are compared with DOA^20^, QBFO^22^ and TSG^23^ that are widely used in this field to verify the feasibility and efficiency of the proposed methods. The performance of the four algorithms is compared by using the standard test function Rastrigrin^24^ as the fitness function, as shown in Formula (17):

17 $$f{(}y{)} = \sum\limits_{i = 1}^{20} {[y_{i}^{2} - 10\cos 2\pi y_{i} + 10]}$$

$$\text{y}_{i} \in {[ - 100,100]}.$$

**Figure 2:**
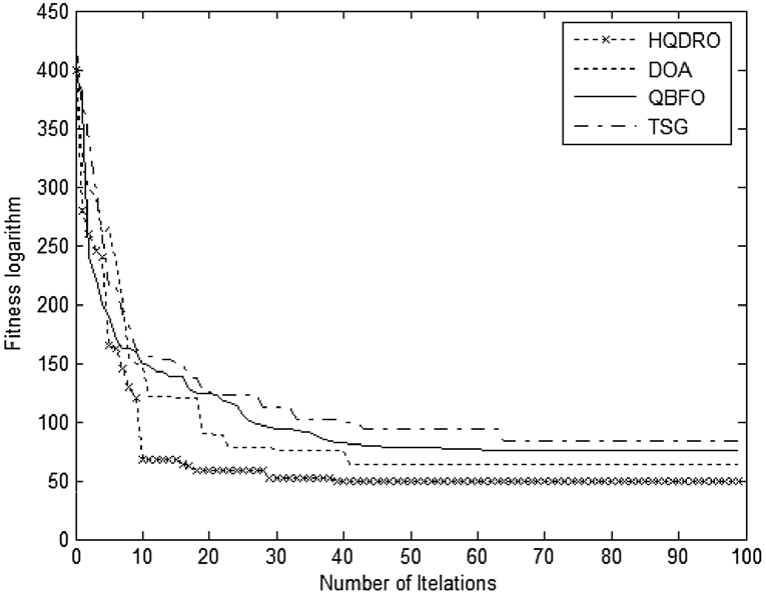
The fitness convergence curve of 4 algorithms.

Set the number of individual dandelions to 20 and the maximum number of iterations to 100. It shows the convergence curve of the approximate optimal solution for the test function operation using four algorithms in Fig. 2. From Fig. 2, this paper presents that the convergence speed and fitness value of HQDRO algorithm are significantly higher than other algorithms, and it has better global optimization ability.

## Example simulation

In order to verify the effectiveness of HQDRO method based on prospect theory, the experimental data set in this section are as follows: A COVID-19 Department has been isolated from the epidemic area as a control area. The new crown pneumonia epidemic prevention and control headquarters has established 46 Epidemic relief point (ERP) in the region. The abscissa and ordinate of each rescue point are randomly generated within [0,60], (unit: km). Specific data are shown in Table 1. The materials needed for epidemic prevention and control are urgently gathered from regional foreign exchange to PIVAS, set coordinates to (30,30). These materials need to be delivered to each ERP as soon as possible; The quantity of materials allocated for each rescue point is generated according to the demand weight in the interval [200,600].

**Table 1:** ERP coordinates and distribution of medical materials.

ERP	Abscissa	Ordinate	Allocation	ERP	Abscissa	Ordinate	Allocation
1	37.8	34.8	493	24	13.8	54.2	332
2	1	25.8	362	25	29.8	42.4	529
3	56.2	54.6	303	26	5.4	10.2	314
4	31.2	13.8	523	27	45.6	39	441
5	32.8	40.8	211	28	11.8	12.4	468
6	41.8	39.6	507	29	56.2	39.4	517
7	19.2	8.2	501	30	14.2	54.8	347
8	24.6	32.2	481	31	58.2	4.6	494
9	25.6	8.2	314	32	33	11.8	524
10	16.6	22	413	33	51	43.6	254
11	9.6	53	458	34	34	35.8	570
12	18.6	8.4	512	35	55	3.6	348
13	11.8	42	368	36	19.8	27	501
14	15.6	14.2	585	37	37.2	10.2	524
15	26.4	22.2	465	38	58	53.8	441
16	39	32.4	590	39	10.8	22.2	293
17	33.4	42	351	40	46.8	56.8	271
18	58	25	281	41	2.8	15	461
19	46.6	8.8	555	42	3.4	34	516
20	2.4	39	417	43	29.4	14.4	595
21	16	40	206	44	37	34.6	443
22	31.2	46	323	45	0.4	19	309
23	37.2	33.6	407	46	19.2	55.2	293

### DRO based results

#### Selection and division of transit point (TP) based on DRO

**Figure 3:**
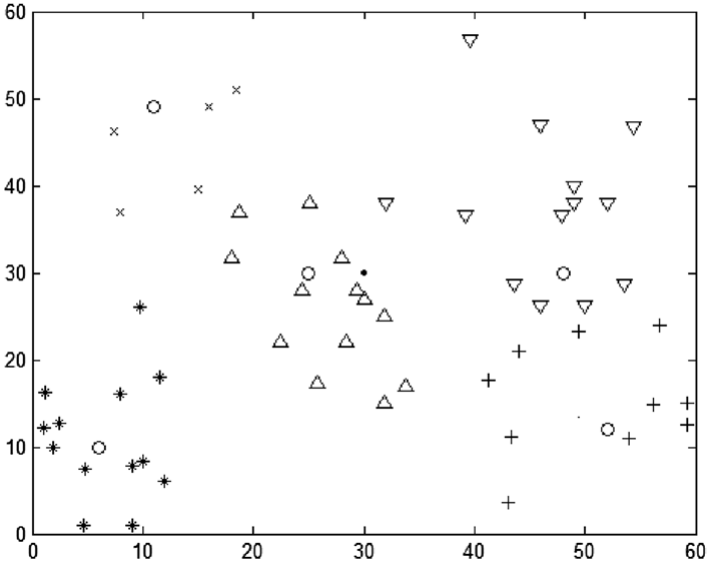
TP selection and division based on DRO.

This paper implements the DRO algorithm in 4.1 based on MATLAB R2018a. The relevant parameters of the algorithm are set as follows: DRO fuzzy weighting coefficient w = 1.5, iterative algorithm termination threshold = 1 × 10^–5^, and the maximum number of iterations is set to 100. After 38 iterations, the algorithm reaches the termination condition, and the final DRO objective function value is 6975.58. The results of TP selection and ERP division are shown in Fig. 3 (the box represents PIVAS, five circles represent the selected TP, and the other five different shapes represent ERP).

Table 2 lists the specific location of TP selected and the ERP cluster responsible for distribution at each transfer point. The vehicle residual capacity (VRC) of each transfer point Distribution area (DA) can be obtained by combining the material allocation of each ERP in Table 1. In Table 2, N^Ci^ represents the adjusted medical assistance point DA, n^Ci^ represents the number of assistance points, Ms_j_ represents the material allocation of assistance point j, and C_rs_ represents VRC. As can be seen from Table 2, there is more residual capacity in DA1 to DA4. Although the DRO method minimizes the total distance between the emergency transfer point and the medical assistance point, because the DRO method only considers the distance criterion and lacks the vehicle capacity constraint for each DA, it is easy to lead to the imbalance of VRC in the transfer point.

**Table 2:** Selection and division of TP based on DRO.

DA	Abscissa	Ordinate	$N^{{C_{i} }}$	$n^{{C_{i} }}$	$Ms_{j}$	$C_{rs}$
*P*_1_	52.13	12.46	{1,7,12,18,22,27,36,37,40}	9	242	12.23
*P*_2_	6.33	10.15	{4,8,9,11,19,20,25,30,32,39,45}	11	239	16.52
*P*_3_	48.02	30.19	{2,5,10,13,15,17,26,28,31,33,41,44,46}	13	347	11.16
*P*_4_	25.08	30.16	{3,14,16,23,24,29,35,42}	8	221	14.84
*P*_5_	11.08	49.25	{6,21,34,38,43}	5	114	4.56

#### DRO based distribution route results

**Figure 4:**
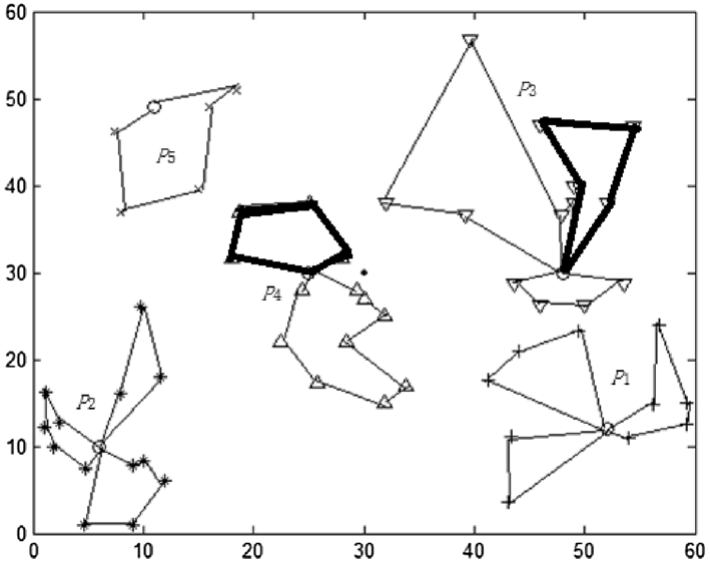
Distribution route selected by TP based on DRO.

After the TP location is selected, the subsequent problem is to plan the transportation route from the transfer point to ERP and design the optimization model of transportation route. It is assumed that the vehicle capacity is 20 m^3^ and the vehicle running speed is 80 km / h. The route structure is shown in Fig. 4 and Table 3: The total delivery time is 633.82 min, the number of medical assistance vehicles is 12, the total VRC is 59.31 m^3^, the average waiting time is 52.82 min, and the maximum waiting time is 72.53 min.

**Table 3:** Distribution route of transfer point based on DRO.

DA	Distribution route	VRC (m^3^)	Total waiting time of materials		Maximum waiting time of materials	
			PIVAS → TP	TP → ERP	PIVAS → TP	TP → ERP
*P*_1_	0 → 37 → 1 → 0	1.06	32.93	48.75	32.93	48.71
	0 → 22 → 36 → 40 → 0	3.86	32.53	112.35	32.53	72.53
	0 → 12 → 18 → 7 → 27 → 0	7.31	32.93	90.11	32.93	56.53
*P*_2_	0 → 4 → 19 → 32 → 9 → 0	12.32	25.92	176.15	25.92	59.58
	0 → 11 → 30 → 25 → 20 → 8 → 0	3.56	25.92	140.98	25.92	44.01
	0 → 39 → 45 → 0	0.45	25.92	43.97	25.92	38.01
*P*_3_	0 → 15 → 10 → 26 → 2 → 31 → 0	8.32	37.36	119.12	37.36	62.16
	0 → 33 → 17 → 13 → 41 → 44 → 0	0.99	37.36	83.77	37.36	52.43
	0 → 46 → 5 → 28 → 0	1.85	37.36	103.05	37.36	41.64
*P*_4_	0 → 29 → 35 → 3 → 0	12.11	36.32	59.8	36.32	39.32
	0 → 14 → 23 → 24 → 16 → 42 → 0	2.73	36.32	113.16	36.32	55.15
*P*_5_	0 → 38 → 21 → 34 → 43 → 6 → 0	4.56	39.29	123.42	39.29	63.75

From the above results, it can be seen that the DRO method can obtain the TP division location and the corresponding material transportation route, but due to the lack of constraints on VRC in the division, the division is unreasonable. For example, the route marked by the thick line in Fig. 4 has large vehicle capacity idle, and the remaining capacity is 12.32 and 12.11 respectively. Other lines also have different degrees of remaining capacity.

### Results based on HQDRO

#### Selection and division based on HQDROTP

**Figure 5:**
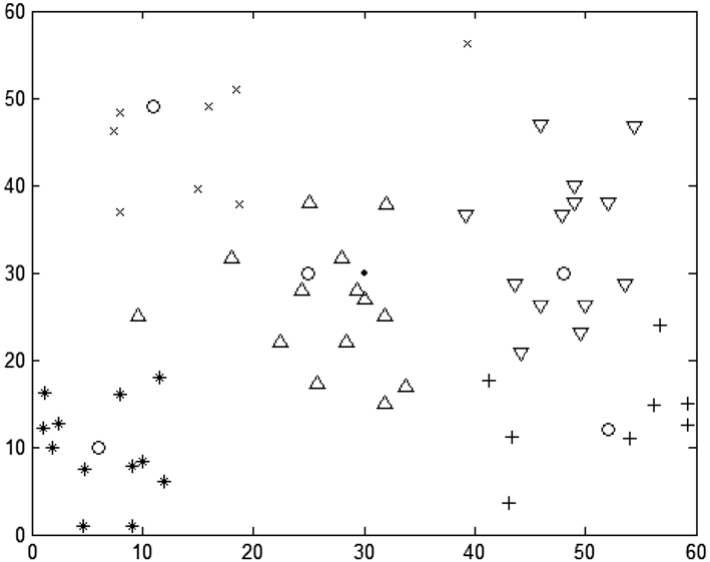
Division of configuration transfer points based on HQDRO.

Considering the limitations of DRO method, this section continues to run Matlab R2018a to realize the HQDRO algorithm in 4.1. The final HQDRO adjustment results are shown in Fig. 5 and Table 4. By comparing Tables 2 and 4, it can be found that the adjustment of the division of epidemic relief points by HQDRO reduces the VRC in each division.

**Table 4:** Division of configuration transfer points based on HQDRO.

DA	Abscissa	Ordinate	$N^{{C_{i} }}$	$n^{{C_{i} }}$	$Ms_{j}$	$C_{rs}$
*P*_1_	52.13	12.46	{1,7,12,18,22,27,36}	7	209	12.42
*P*_2_	6.33	10.15	{4,8,9,11,19,20,25,30,32,39}	10	224	10.17
*P*_3_	48.02	30.19	{2,5,10,13,15,17,26,28,31,33,37,40,41}	13	349	7.34
*P*_4_	25.08	30.16	{3,14,16,23,24,29,35,44,45}	9	232	8.41
*P*_5_	11.08	49.25	{6,21,34,38,42,43,46}	7	153	1.50

#### Transportation route based on HQDRO

**Figure 6:**
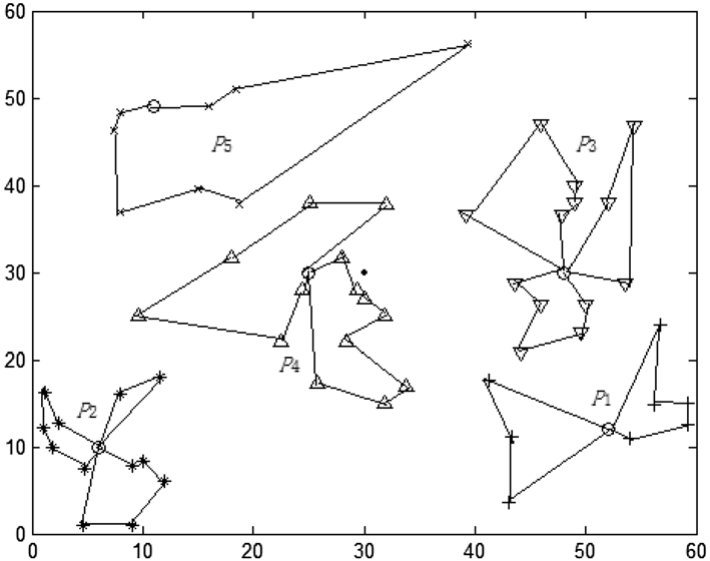
Transit point distribution route based on HQDRO.

The same parameters as those set in 4.1: the vehicle capacity is 20 m^3^ and the vehicle running speed is 80 km/h. The calculation results are shown in Fig. 6 and Table 5: the total delivery time is 511.77 min, the number of epidemic relief vehicles is 11, and the total VRC is 39.84 m^3^, which is nearly 20 m^3^ less than the DRO method (the capacity of one vehicle); The average waiting time was 46.52 min and the maximum waiting time was 67.23 min.

**Table 5:** Transit point distribution route based on HQDRO.

DA	Distribution route	VRC (m^3^)	Total waiting time of materials		Maximum waiting time of materials	
			PIVAS → TP	TP → ERP	PIVAS → TP	TP → ERP
*P*_1_	0 → 1 → 36 → 12 → 0	6.36	29.87	45.69	29.83	43.41
	0 → 18 → 22 → 27 → 7 → 0	6.06	29.47	109.29	29.43	67.23
*P*_2_	0 → 4 → 19 → 32 → 9 → 0	2.59	29.87	87.05	29.83	51.23
	0 → 11 → 30 → 25 → 20 → 0	3.07	22.86	173.09	22.82	54.28
	0 → 8 → 39 → 0	4.51	22.86	137.92	22.82	38.71
*P*_3_	0 → 15 → 10 → 26 → 2 → 31 → 0	2.38	22.86	40.91	22.82	32.71
	0 → 33 → 17 → 13 → 41 → 0	1.14	34.3	116.06	34.26	56.86
	0 → 37 → 40 → 5 → 28 → 0	3.82	34.3	80.71	34.26	47.13
*P*_4_	0 → 29 → 35 → 3 → 45 → 0	6.17	34.3	99.99	34.26	36.34
	0 → 14 → 23 → 44 → 24 → 16 → 0	2.24	33.26	56.74	33.22	34.02
*P*_5_	0 → 38 → 21 → 34 → 42 → 6 → 43 → 46 → 0	1.50	33.26	110.1	33.22	49.85

### Comparative analysis

In order to verify the effectiveness of the proposed HQDRO, the researcher compared the results of the proposed method with those obtained using the DRO method^20^ in terms of Number of vehicle (NV), Total VRC (TV), Maximum Single VRC (MSV), Average waiting time (AT) and Maximum waiting time (MT) respectively, as shown in Table 6.

**Table 6:** Comparison of DRO and HQDRO.

Method	NV	TV	MSV	AT	MT
DRO	12	59.31	12.32	52.82	72.53
HQDRO	11	39.84	6.36	46.52	67.23

From the comparison results in Table 6, it can be seen that for this paper 46 ERPs are proposed for the distribution transit requirements:

1. From the NV point of view, the HQDRO method uses 11 delivery vehicles while minimizing undelivered time as much as possible, whereas the DRO method runs results in 12 vehicles.
2. From the TV point of view, the HQDRO distribution process vehicle residual capacity is 32.83% lower than the vehicle residual capacity of the DRO method.
3. From the MSV point of view, the maximum remaining capacity of a single vehicle with HQDRO is 6.36m3, which is more than 40% more than the maximum remaining capacity of a single vehicle with DRO of 12.32m3; the combined TV and MSV show the advantage of this method in terms of fully utilizing vehicle capacity.
4. From the AT and MT point of view, the time savings with HQDRO versus DRO are 11.93% and 7.31% respectively, making this method superior to the DRO method.

In summary, the proposed HQDRO minimizes the number of vehicles dispatched as well as the space and time costs of distribution compared to the general DRO method. Therefore, the results obtained by this method are more practical.

## Conclusion

Aiming at the problems of improper allocation of medical supplies between medical supplies and epidemic relief points, low allocation efficiency, and a high vacancy rate of distribution vehicles that may occur in sudden epidemic disasters, a two-stage robust allocation model for medical supplies and an HQDRO algorithm based on the two-stage decision model are proposed by introducing prospect theory. After comparing with three commonly used algorithms in this field, it is verified that the convergence speed and fitness value of the proposed HQDRO algorithm are significantly higher than those of other algorithms, and it has a better global optimization finding ability. This paper was run with 46 ERPs in a region closed to control due to an epidemic, and the following main conclusions were drawn from the results. (1) The general DRO algorithm results in a division that minimizes the total distance between the configured transit point and the medical aid point, but the lack of constraints on the capacity of the vehicles in each division results in a large residual capacity (vacancy rate); (2) The adjustment of the medical aid point divisions by HQDRO makes the VRC in each division smaller and enables a reduction in the total delivery time and average waiting time, effectively reducing the number of delivery vehicles. Based on the results of this paper, the next step in the research is to accurately predict the number of medical supplies needed at the aid points based on the epidemic scenario and how to overcome the unevenness in the number of aid points.

## Data Availability

The datasets generated during the current study are not publicly available due reason for confidentiality but are available from the corresponding author on reasonable request.
